# Identification of a natively resilient but poorly regenerating retinal ganglion cell type in the G protein-coupled receptor 88-Cre transgenic mouse

**DOI:** 10.4103/NRR.NRR-D-24-01270

**Published:** 2025-08-13

**Authors:** Allison L. Hall, Christopher Zhao, Sean McCracken, Minglei Zhao, Zelun Wang, Andrea Santeford, Rajendra S. Apte, Philip R. Williams

**Affiliations:** 1 John F. Hardesty, MD Department of Ophthalmology and Visual Sciences, Washington University School of Medicine, St. Louis, MO, USA; 2 Graduate Program in Neuroscience, Washington University School of Medicine, St. Louis, MO, USA; 3 Medical Scientist Training Program, Washington University School of Medicine, St. Louis, MO, USA; 4 Department of Developmental Biology, Washington University in St. Louis School of Medicine, St. Louis, MO, USA; 5 Department of Medicine, Washington University in St. Louis School of Medicine, St. Louis, MO, USA; 6 Department of Neuroscience, Washington University School of Medicine, St. Louis, MO, USA; 7 Hope Center for Neurological Disorders, Washington University School of Medicine, St. Louis, MO, USA

**Keywords:** axon regeneration, G protein-coupled receptor, neuroprotection, optic nerve injury, retina, retinal ganglion cells

## Abstract

Retinal ganglion cells are susceptible to neurodegenerative conditions and their death drives common forms of irreversible vision loss. In mice, there are 46 transcriptionally unique retinal ganglion cell types that demonstrate different susceptibilities to degeneration. Recent transcriptional experiments defined a novel retinal ganglion cell type that survives particularly well and uniquely expresses high levels of the orphan G-protein-coupled receptor 88. Motivated to study this retinal ganglion cell type, we obtained GPR88-Cre transgenic mice to identify the novel well-surviving retinal ganglion cells and examine their survival and regenerative potential. Our experiments demonstrate that this unidentified retinal ganglion cell type is likely accordant with previously described ON-direction-selective retinal ganglion cells. Interestingly, we find that ON-direction-selective retinal ganglion cells are resilient, but demonstrate limited potential to regenerate their axons in response to well-characterized regenerative treatments. Studying the molecular properties of the ON-direction-selective retinal ganglion cells could unlock new therapeutics to preserve retinal ganglion cells in patients.

## Graphical abstract

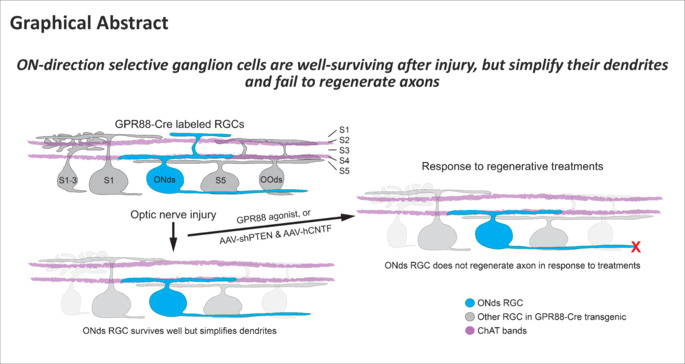

## Introduction

Retinal ganglion cell (RGC) degeneration drives vision loss in common blinding conditions including glaucoma and traumatic optic nerve injury (Resnikoff et al., 2004). RGCs are projection neurons represented by 20 to 45 unique types depending on the species (Tran et al., 2019; Hahn et al., 2023). Importantly, RGC types display vastly different vulnerabilities and resiliency in the face of neurodegeneration in species ranging from human to mouse. For example, large soma RGCs are more vulnerable in primate models of glaucoma (Glovinsky et al., 1991), and large caliber RGC axons preferentially degenerate in human glaucoma patients (Kerrigan-Baumrind et al., 2000).

RGC type susceptibility has been extensively examined in the mouse optic nerve crush (ONC) model of degeneration. Studies using immunostaining and transgenic mouse lines to identify RGC types demonstrated that alpha and intrinsically photosensitive RGCs (αRGCs and ipRGCs respectively) are well-surviving, while W3-RGCs, F-RGCs, and ON-OFF direction-selective RGCs (OOdsRGCs) as susceptible (Duan et al., 2015; Daniel et al., 2018; Tran et al., 2019). Single-cell RNA sequencing identified 46 types of RGCs and tracked their individual survival rates after ONC, demonstrating that RGC type specific survival exists on a continuum from ON-sustained αRGCs (> 80% survival) to F-midi OFF RGC (< 2% survival; Tran et al., 2019). Importantly, only a handful of RGC types are more resilient than the population at large, and few demonstrate survival above 50% including ON-sustained αRGCs, M1 and M2 ipRGCs, and a previously undefined population termed C10, which is marked by high expression of the orphan receptor G-protein-coupled receptor 88 (GPR88) (Tran et al., 2019). Subsequent studies pairing electrophysiology and RNA sequencing suggest that the C10 RGCs could be ON direction-selective (ONdsRGCs) (Goetz et al., 2022; Huang et al., 2022). We were motivated to investigate the C10 RGCs, and thus examined RGCs labeled in a GPR88-Cre transgenic mouse retina (Tran et al., 2019).

Examination of the cellular traits of well-surviving RGCs has identified treatments that protect and regenerate RGCs (Duan et al., 2015; Bray et al., 2019; Tran et al., 2019; Jacobi et al., 2022). Comparing transcriptional profiles of resilient and susceptible RGC types led to the discovery that the urocortin/corticotropin-releasing hormone signaling axis protects and regenerates RGCs (Tran et al., 2019). Osteopontin is highly expressed in well-surviving RGCs, and treatment of RGCs and corticospinal neurons with osteopontin and growth factors increases survival and regeneration in axon injury models (Duan et al., 2015). ipRGCs natively survive well, especially M1 and M2 types that express higher levels of melanopsin (Li et al., 2016; Daniel et al., 2018; Bray et al., 2019; Tran et al., 2019). Adeno-associated virus (AAV)-mediated overexpression of melanopsin, and in fact other Gαs signaling GPCRs, protects RGCs from degeneration after ONC (Li et al., 2016; Bray et al., 2019). Taken together, a better understanding of the cellular traits, survival mechanisms, and regenerative capacity of C10 RGCs could lead to novel treatments to preserve and possibly restore RGCs from degenerative conditions. In this study, we report on the utility of the GPR88-Cre BAC transgenic mouse line for examining the survival and regenerative potential of *GPR88*-expressin RGCs.

## Methods

### Animals

All experimental procedures were performed in accordance with animal protocols approved by the International Animal Care and Use Committee at Washington University in St. Louis Medical School (approval Nos. 20190034 and 22-0043) on April 18, 2019 and March 23, 2022 and in compliance with the NIH Guide for the Care and Use of Laboratory Animals. Male and female mice were used dependent on litters and separated in cohorts of 3–5 siblings. For transgenic lines Ai9 (Jackson Laboratory, Bar Harbor, ME, USA, RRID: IMSR_JAX:007909), Slc17a6-IRES-Cre (Jackson Laboratory, RRID: IMSR_JAX:028863), and GPR88-Cre (Mutant Mouse Resource and Research Center, Davis, CA, USA, RRID: MMRRC_036078-UCD) mouse lines were obtained. The GPR88-Cre BAC transgenic was reconstituted from frozen sperm maintained in the Crl:CD1(ICR) genetic background by *in vitro* fertilization using C57Bl/6J wt oocytes. Progeny were backcrossed to wt C57Bl/6J for a minimum of six generations. Mice for experiments were aged approximately 4–6 weeks prior to AAV injections and injuries were performed on mice at 6–8 weeks of age. Male and female mice were housed one to five per cage in a SPF barrier facility at Washington University School of Medicine with 12-hour light/dark cycles at 21°C and 50% humidity.

### *In situ* hybridization

RNAscope Multiplex Fluorescent V2 assay (ACDBio, Newark, CA, USA, Cat# 323100) was performed according to the manufacturer’s instructions. Eyes were enucleated and globes were drop-fixed for 30 minutes in 4% paraformaldehyde in 1× phosphate-buffered saline (PBS). Fix globes were embedded in paraffin, sectioned at 4 μm thickness, and directly mounted on microscope slides. Slides with eye sections were baked for 60 minutes at 60°C before being deparaffinized in two changes of xylenes and dehydrated in two changes of 100% ethanol for 5 minutes each, then fully dried in a 60°C oven, **~**10 minutes. Slides were then treated with RNAscope hydrogen peroxide solution for 10 minutes at room temperature and washed twice in distilled water prior to 15 minutes of antigen retrieval in 100°C RNAscope target retrieval solution (ACDBio, Cat# 322000). After rinsing in distilled water, slides were dried in 100% ethanol and placed in a 60°C oven for 5 minutes. Barriers were drawn around tissues using an ImmEdge hydrophobic Pen (Vector Labs, Newark, CA, USA, Cat# H-4000) and then slides were allowed to dry at room temperature overnight. The following day, all incubations were performed in a HybEZ oven (ACDBio, Cat# 241000ACD) at 40°C. First, RNAscope Protease II solution (ACDBio, Cat# 322340) was added to each slide for 30 minutes. After two washes in nuclease-free water, probes were applied for 2 hours. tdTomato-C2 probe (ACDBio, Cat# 317041-C2) was diluted 1:50 into ready-to-use solution of probe MM-Gpr88-C1 (ACDBio, Cat# 317451) prior to use. Following the RNAscope Multiplex Fluorescent V2 assay kit’s three amplification solution incubations at and washes in RNAscope wash buffer (diluted to 1× in nuclease-free water), HRP-C1 solution was applied for 15 minutes at 40°C, followed by two changes of wash buffer and application of Opal 520 (Akoya Biosciences, Marlborough, MA, USA, Cat# FP1478001KT) diluted 1:1500 in TSA buffer for 30 minutes at 40°C. After washing, HRP-C2 solution was applied for 15 minutes at 40°C, followed by two additional washes and application of Opal 690 (Akoya Biosciences, Cat# FP1497001KT) for 15 minutes at 40°C. Slides were washed, nuclei were stained with 5 µg/mL DAPI (Millipore-Sigma, Burlington, MA, USA, Cat# D9542) for 20 minutes at room temperature, washed in wash buffer twice and cover slipped using FluoroSave Reagent (Millipore-Sigma, Cat# 345789).

### Tissue collection and immunostaining

Animals were given an intraperitoneal overdose injection of Tribromoethanol (Avertin, 500 mg/kg) (Sigma, St. Louis, MO, USA, Cat# T48402) and perfused with ice-cold PBS followed by 100 mL of 4% paraformaldehyde (Sigma) in PBS, or killed by cervical dislocation and retinas dissected directly in 4% paraformaldehyde. Retina wholemounts were sunk overnight in 30% sucrose in PBS at 4°C and freeze-thawed three times using dry ice and a glass slide for permeabilization. Samples were washed with PBS three times for 10 minutes and put in blocking solution (10% normal horse serum (Sigma, Cat# 158127) and 0.5% Triton X-100 (Sigma, Cat# 11332481001) in PBS) for 1–3 hours at room temperature. Samples were placed in a solution of primary antibodies diluted in blocking solution on a shaker at 4°C for 5–7 days. Primary antibodies used were the following: guinea pig anti-RNA binding protein with multiple splicing (Rbpms; 1:2000, Raygene, Hangzhou, China, Cat# A008712), mouse anti-activator protein 2 (AP2; 1:100, Developmental Studies Hybridoma Bank, Iowa City, IA, USA, Cat# 3B5), goat anti-choline acetyltransferase (ChAT; 1:200, Millipore, Cat# AB144P), goat anti-osteopontin (Spp1; 1:600, R&D Systems, Minneapolis, MN, USA, Cat# AF1433, RRID: AB_354791), chicken anti-Tbr2 (1:2000, Millipore, Cat# AB15894, RRID: AB_10615604), and rabbit anti-CART (1:1500, Phoenix Pharmaceuticals, Burlingame, CA, Cat# H-003-62, RRID: AB_2313614). Retinas were washed with PBS three times for 10 minutes at room temperature. Secondary antibodies (all from Jackson ImmunoResearch, West Grove, PA, USA) were diluted in blocking solution for 2–3 days at 4°C. Secondary antibodies were raised in donkey against the primary antibodies host species, cross absorbed and conjugated to Alexa Fluor 488 (Cat# 703-545-155, RRID: AB_2340375; Cat# 706-545-148, RRID: AB_2340472; Cat# 715-545-150, RRID: AB_2340846; Cat# 705-545-147, RRID: AB_2336933), 568 (Cat# 705-575-147, RRID: AB_3095466; Cat# 706-575-148, RRID: AB_3095468) or 647 (Cat# 703-605-155, RRID: AB_2340379; Cat# 711-605-152, RRID: AB_2492288; Cat# 706-605-148, RRID: AB_2340476), and used at 1:500–1000 dilution. After washing three times in PBS, whole retinas were mounted onto glass slides with Vectashield Antifade Mounting Medium (Vector Labs, Cat# H-1000-10).

Brains were collected from transcardiacally perfused mice cut into appropriate slabs that were then cryoprotected in 30% sucrose in PBS overnight at 4°C. Brain slabs were embedded in optimal cutting temperature compound (OCT; Scigen, Paramount, CA, USA, Cat# 4586), and cryosectioned at 40 μm into PBS. Free-floating sections were stained as above for retinal wholemounts except primary antibodies were only incubated overnight at 4°C, and secondary antibodies for 3 hours at room temperature. Mouse anti-NeuN (1:300, Millipore, Cat# MAB377B, RRID: AB_177621) was used to counterstain brain sections. Free-floating sections were mounted onto slides and coverslipped with Vectashield Antifade Mounting Medium.

Optic nerves were collected and cryoprotected by incubation in 15% sucrose in PBS overnight at 4°C. After embedding in OCT, nerves were cut longitudinally in series onto slides at 12 μm thickness using a cryostat.

### Intravitreal adeno-associated virus injections

To sparsely label GPR88-Cre RGCs and to measure cytoplasmic calcium levels, we used AAVs carrying Brainbow fluorescent reporters (Addgene, Watertown, MA, USA) or the Ca^2+^ biosensor Twitch2b (Hope Center Viral Vectors Core, St. Louis, MO, USA) respectively. To induce axon regeneration AAV2 carrying recombinant human ciliary neurotrophic factor (CNTF) and/or shorthairpin RNA against PTEN with a green fluorescent protein reporter were used. AAV Brainbow aliquots were purchased from Adgene (AAV-EF1a-BbTagBY, 45185, AAV-Ef1a-BbChT, 45186 (Cai et al., 2013)) and AAV2-FLEX-Twitch2b (McCracken et al., 2023), AAV2-hCNTF and AAV-shPTEN (Smith et al., 2009; Sun et al., 2011; Zukor et al., 2013) were packaged into AAV2 by The Hope Center Viral Vectors Core at Washington University in St. Louis. The titers of viral preparations ranged from 2 × 10^12^ to 1.0 × 10^13^ GC/mL as measured by qPCR. The virus was stored in 10 or 20 µL aliquots in a –80°C freezer.

Mice were anesthetized by intraperitoneal injection of a ketamine and xylazine cocktail (KX) (10 mg/mL and 1 mg/mL, respectively) (Department of Comparative Medicine at Washington University, St. Louis, MO, USA) in saline at a dose of 10 µL/g body weight. A pulled-glass micropipette was inserted near the peripheral retina behind the ora serrata and deliberately angled to avoid damage to the lens. Approximately 1 mL of vitreous humor was removed prior to viral injection. Virus aliquots were centrifuged for at least 30 seconds prior to injection to remove air bubbles. Between 1.5–2 µL of AAV was injected intravitreally using a Hamilton syringe (80950, Hamilton Company, Reno, NV, USA). Any injections resulting in air within the vitreous or lens injury were not included in experiments. An anti-bacterial ophthalmic ointment, Terramycin (Zoetis, NADA #8-763) was applied post-operatively to protect the cornea. All animals received subcutaneous Meloxicam (10 mg/mL) at a dose of 10 µL/g of body weight as a postoperative analgesic.

### Optic nerve crush

Bilateral optic nerve crush (ONC) injury was performed as previously (Park et al., 2008). In brief, mice were anesthetized with KX as above and the optic nerve was exposed intraorbitally and crushed with fine forceps (Fine Science Tools, Foster City, CA, USA, Carbon #5, No. 11251-10) for 10 seconds approximately 500 µm behind the optic disc. Anti-bacterial eye ointment was applied post-operatively to protect the cornea, and animals received subcutaneous Meloxicam (10 mg/mL) at a dose of 10 µL/g of body weight as a postoperative analgesic. Animal health was monitored for the first 2 days after ONC and every other day throughout the 14-day experiment. For *in vivo* imaging experiments, mice received a binocular ONC two days after their pre-image was taken. When evaluating the effect of (1R,2R)-2-PCCA (TargetMol, Boston, MA, USA; T13423) on survival and regeneration, intravitreal injection of 1 μL 237 μM (1R,2R)-2-PCCA diluted in 1 × PBS was first performed immediately after ONC as described above for AAV and at indicated days thereafter. Vehicle controls were 1 μL of PBS. Care was taken to avoid parts of the eye affected by the ONC surgery, where injections were performed at the nasal or dorsal portions of the eye rather than temporally.

### Anterograde retinal ganglion cell axon tracing

Two to three days before perfusion, mice were anesthetized as above using a KX cocktail. Cholera toxin subunit B (CTB; 2 μL) conjugated to Alexa Fluor 647 (1 μg/μL in sterile PBS; ThermoFisher, Waltham, MA, USA) was injected intravitreally with a pulled glass micropipetter attached to a Hamilton syringe as described above for intravitreal AAV injections. Anti-bacterial eye ointment was applied post-operatively to protect the cornea, and animals received subcutaneous Meloxicam (10 mg/mL) at a dose of 10 µL/g of body weight as a postoperative analgesic.

### Confocal microscopy

An inverted laser scanning confocal microscope (Zeiss, Oberkochen, Germany; Model 710) equipped with a 20× air (Zeiss ‘Plan Apochromat’, 0.8 NA, 420650-9902), 40× water immersion, and 63× oil immersion, objectives were used to acquire image stacks with 0.4–1.0 μm z-stepping. Immunostained retinas were imaged for their respective antibodies as well as native fluorophore or CTB fluorescence. For *in situ* hybridization imaging, a 20× objective was used to acquire images of retinal cryosections with 1–2 μm z-steps. For wholemount imaging of cell type markers or RGC survival with Rbpms, a montage of a 4 × 4 tiled field of the retina surrounding the optic nerve head was obtained, the total region being 2100 mm × 2100 mm, and images were automatically stitched with the Zeiss ZEN Blue software (RRID: SCR_013672). Four regions in each cardinal direction were analyzed using the Cell Counter plugin in ImageJ (version 1.54f, National Institutes of Health, Bethesda, MD, USA, RRID: SCR_003070). For high-resolution reconstructions of dendritic arbors, either 40× or 63× oil objectives were used. Confocal stacks from the retina surface to the beginning of the inner plexiform layer were obtained with resolution-limited settings for pixel size and 0.4–0.5 μm z-steps. Cells were scored as being an RGC based on being immunopositive for Rbpms, and/or possessing a clear axon in the nerve fiber layer. Arbor stratification was analyzed against ChAT immunostaining by stepping through the z-planes, examining orthoslice views, and in some instances in Imaris software (version 10.0, Oxford Instruments, Abindgon, UK, RRID: SCR_007370) for 3-D rendering. The inner plexiform layer was divided into 5 strata, S1 above both ChAT bands, S2 the upper ChAT band, S3 between the ChAT bands, S4 the lower ChAT band, and S5 below both ChAT bands. The dendritic field diameter was measured in ImageJ using the line tool. A set of exemplar cells was skeletonized using Imaris software to display depth across the arbor. Brain sections were imaged with a 20× objective 1–2 μm z-steps and montages of full sections or hemisections were acquired and automatically stitched with the Zeiss Blue software. Brain regions were identified by matching NeuN staining to a reference atlas (Paxinos and Franklin, 2013). Optic nerve sections were imaged using a 20× objective with z-stepping set to 1.2 μm. Montages were acquired covering all observable axons and automatically stitched with the Zeiss Blue software. Regenerating axons were scored at indicated distances past the crush site, and for the presence or absence of tdTomato signal manually using the Cell Counter tool in ImageJ.

### *In vivo* Ca^2+^ imaging

*In vivo* imaging was performed as previously described (Wang et al., 2021). A Scientifica Hyperscope (Scientifica, Maidenhead, UK) was used for *in vivo* image acquisition. This consists of a Mai Tai HP 100 fs pulsed laser (Spectra-Physics, Milpitas, CA, USA), a pockels cell to modulate laser power, and a pair of glavo mirrors for beam steering. A 20 mm working distance objective (Mitutoyo, Aurora, IL, USA; 20X air, 0.4 N.A., 378-824-5) was used for the relay of the mouse lens and a motorized objective mount for z-stepping. A Chromoflex light collection system paired with GaAsP photo-multiplier tube detectors (Scientifica) was used to acquire fluorescence emission signals. To visualize the emission signal from the Twitch-2b sensor, we used a filter cube consisting of a 505 long pass dichroic and 480/40 and 535/30 band pass filter pairs to separate the cyan fluorescent protein signal and yellow fluorescent protein (YFP) signal.

Mice were anesthetized with KX cocktail as described above and placed in an imaging head holder (SGM 4, Narishige, Tokyo, Japan) to keep stationary while imaging. A solution of 1% w/v atropine and 2.5% w/v phenylephrine hydrochloride in RO water was applied with an eye dropper to both eyes for pupil dilation, and animals were placed in the dark for 5–15 minutes prior to being put under the objective. Genteal-tears eye ointment (Alcon Inc., Fort Worth, TX, USA) was applied to both eyes and the head of the mouse was angled to align the iris with the light path. A #1.5 coverslip was placed in a compact filter holder (Thorlabs, Newton, NJ, USA; DH1) and the holder was fixed to the microscope stage, allowing for the coverslip to be held perpendicular to the light path centered on the imaging area.

Laser power measured out of the objective ranged from 20–45 mW and was limited to a maximum of 45 mW to prevent retina or cornea damage (Wang et al., 2021). The Mai Tai pulsed laser was set at 850 nm. ScanImage acquisition software (Vidrio Technologies, MBF Bioscience, LLC, Williston, VT, USA; RRID: SCR_014307) was used to obtain image stacks with an 8 mm z-stepping. Images (512 pixels x 512 pixels, 1 pixel ≈ 1 µm^2^) were collected at 0.93 Hz with a 3-frame average. Retinas were scanned from the ganglion cell layer towards the inner nuclear layer to reduce photoreceptor activation. Other imaging settings like photo-multiplier tube voltage, bias voltage, and digital zoom were kept constant across all images.,

Two-photon image analysis was performed using ImageJ software. Cell bodies were identified and manually labeled as individual elliptical regions of interest in their brightest and best-resolved z-section. Mean pixel values within each region of interest for both cyan fluorescent protein and YFP channels were measured in ImageJ and ratios were recorded and interpreted as physiological intracellular Ca^2+^ levels. For chronic survival scoring, image stacks were processed by maximally projecting processed images to a single plane, combining the time series of max-projections into a t-stack multi-tiff, and aligning these images with the Linear Stack Alignment with SIFT plugin in ImageJ. Cells were scored as persisting through the *in vivo* time series or disappearing and considered dead. The presence or absence of tracked cells was confirmed by high-resolution confocal imaging of retinal flat mounts. Tracked cells were confirmed to be RGCs by immunostaining for Rbpms.

### Statistical analysis

Mann-Whitney *U* Test was used for comparisons of two groups. Two-way analysis of variance with Bonferroni correction was used for multigroup, or multi-time point comparisons. Statistics were performed using GraphPad Prism9 Software (Dotmatics, Boston, MA, USA).

## Results

### GPR88-Cre expressing cells in the retina

We first determined the fidelity of the GPR88-Cre BAC transgenic line by crossing GPR88-Cre and Ai9 reporter transgenic mice to convey Cre-dependent tdTomato expression in all GPR88-Cre positive cells. We performed *in situ* hybridization for *tdTomato* and GPR88, and demonstrated that the GPR88-Cre transgenic line was faithful to native *GPR88* transcript expression in the retina (**Additional Figure 1**). Specifically, 100% of *GPR88*-positive cells were positive for *tdTomato*, while 100% of *tdTomato*-positive cells were also positive GPR88 (87 cells from 6 retinas).

**Figure 1:**
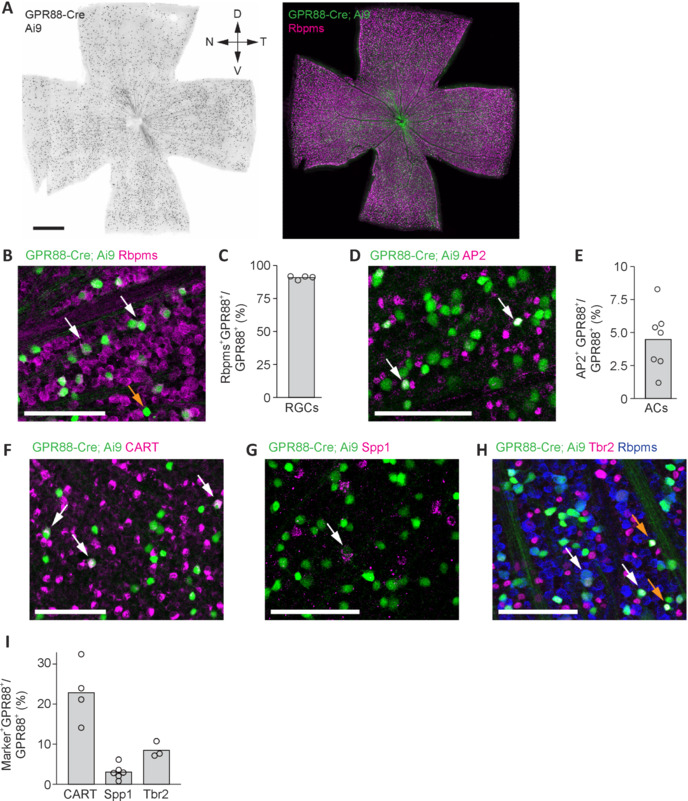
Cell types labeled by GPR88-Cre. (A) Confocal maximum intensity projection of a GPR88-Cree x Ai9 retinal wholemount (gray left, green right) immunostained for Rbpms (magenta right). Scale bar: 500 μm. D: Dorsal; N: nasal; T: temporal; V: ventral. (B) Confocal maximum intensity projection of GPR88-Cre x Ai9 (green) retinal wholemount immunostained for Rbpms (magenta). White arrows indicate Rbpms-positive cells, orange arrows indicate Rbpms-negative cells. (C) Quantification of the proportion of GPR88-Cre x Ai9 cells that were Rbpms positive (*n* = 13,138 cells from 4 retinas). (D) Confocal maximum intensity projection of GPR88-Cre x Ai9 retinal wholemount immunostained for AP2 (magenta). (E) Quantification of the proportion of GPR88-Cre x Ai9 cells that were AP2 positive (*n* = 5336 cells from 7 retinas). (F, G) Confocal maximum intensity projection of GPR88-Cre x Ai9 retinal wholemount immunostained for CART and Spp1 (magenta) respectively. (H) Confocal maximum intensity projection of GPR88-Cre x Ai9 (green) retinal wholemount immunostained for Rbpms (blue) and Tbr2 (magenta). White arrows indicate cells positive for Tbr2 and Rbpms. Orange arrows indicate likely amacrine cells only positive for Tbr2. Scale bars: 100 μm. (I) Quantification of the proportion of GPR88-Cre x Ai9 cells that were CART, Spp1, or Tbr2 (and Rbpms) positive (*n* = 2275 RGCs from 4 retinas, *n* = 3552 RGCs from 6 retinas, and 754 RGCs from 3 retinas respectively). Bars represent mean of samples, circles are individual retinas counted in 3–4 regions. GPR88: G-protein-coupled receptor 88; RGCs: retinal ganglion cells.

Observing the distribution of GPR88-Cre positive cells in the Ai9 reporter cross retina showed that cells were qualitatively less dense in the dorsal retina, and also underrepresented in the temporal retina (**Figure 1A**) where a high density of specific RGC types mediate high binocular vision (Johnson et al., 2021). Since GPR88 expression has been reported by single-cell RNAseq in both amacrine cells and RGCs, we wanted to determine if GPR88-Cre labeled cells in the ganglion cell layer were all RGCs. To demonstrate the distribution of RGCs and amacrine cells in the GPR88-Cre; Ai9 retina, we immunostained wholemount retinas with Rbpms and AP-2 antibodies, respectively. We found that most Cre-expressing neurons in the ganglion cell layer of the retina were RGCs (90.8% ± 0.7% Rbpms positive), with a small fraction of GPR88 retinal neurons being amacrine cells (4.5% ± 0.9% AP2 positive; **Figure 1B–E**). We observed no cells in the retina that were negative for both AP2 and Rbpms. 11.6% ± 1.0% of Rbpms labeled RGCs were positive for reporter expression, suggesting that multiple types of RGCs may express Cre in the GPR88-Cre transgenic retina.

To identify RGC types that are labelled in the GPR88-Cre reporter line, we first performed immunostaining for certain RGC type markers (**Figure 1F–I**) including osteopontin (Spp1) for αRGCs (ipRGCs; Duan et al., 2015)), Tbr2 for intrinsically photosensitive RGCs (ipRGCs; Chen et al., 2021), or CART for OOdsRGCs (Kay et al., 2011), along with Rbpms to verify analyzed cells were RGCs. We observed Spp1 labeled (3.0% ± 0.7%), Tbr2 positive RGCs (8.5% ± 1.1%), and CART positive (22.9% ± 11.5%) RGCs in GPR88-Cre x Ai9 transgenic retinas. The enrichment for OOdsRGCs as labeled by CART is in accordance with *in situ* hybridization for *GPR88* localizing to RGCs with OOdsRGC morphology (Tran et al., 2019).

**Figure 2:**
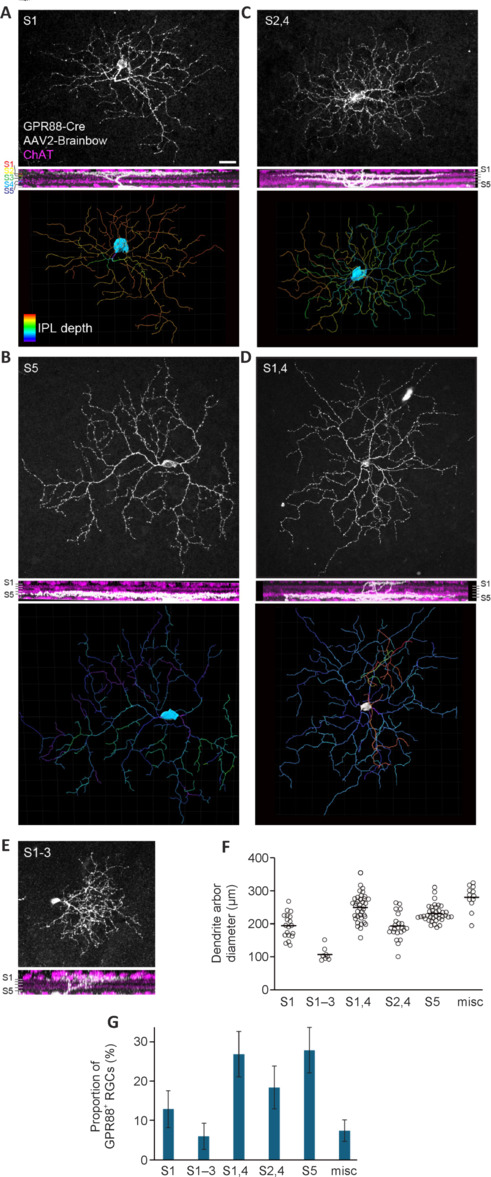
Representative morphology of RGC types labeled by the GPR88-Cre transgenic line. (A) Confocal maximum intensity projection (upper), orthogonal projection (middle), and skeletonization (lower) of RGC projecting to layer S1 of the IPL. AAV-Brainbow (grayscale), ChAT (magenta), skeleton pseudocolored according to depth in the confocal stack volume. Scale bar: 20 μm. (B) Confocal maximum intensity projection (upper), orthogonal projection (middle), and skeletonization (lower) of RGC projecting to layer S5 of the IPL. (C) Confocal maximum intensity projection (upper), orthogonal projection (middle), and skeletonization (lower) of RGC projecting to layers S2 & S4 of the IPL. (D) Confocal maximum intensity projection (upper), orthogonal projection (middle), and skeletonization (lower) of RGC projecting to layer S1 & S4 of the IPL. (E) Confocal maximum intensity projection (upper), lower orthogonal projection (lower) of RGC projecting to layers S1–S3 of the IPL. (F) Scatterplot of isolated RGC arbor diameters measured from confocal image stacks (*n* = 138 RGCs from 26 retinas). (G) Proportion of each type of RGC identified by low-efficiency AAV labeling of GPR88 transgenic retinas (*n* = 200 RGCs from 26 retinas). Error bars are 95% confidence interval. ChAT: Choline acetyltransferase; GPR88: G-protein-coupled receptor 88; RGCs: retinal ganglion cells.

To further define specific RGC types labeled in the GPR88-Cre transgenic retina, we reconstructed individual RGCs labelled using low-density AAV2-Brainbow methods (200 cells from 26 retinas; **Figure 2** (Cai et al., 2013)). Isolated RGCs were confirmed by the presence of an axon and/or Rbpms immunostaining and qualitatively categorized based on their arbor size, density, and stratifications in the inner plexiform layer (IPL). We identified five morphologically-distinct types of RGCs that were reliably represented, each of which had a unique stratification pattern in the IPL. The most commonly observed RGC type (28.0%; 95% Confidence Interval (CI): 22.2%–33.78%) in the GPR88-Cre line was a medium-field monostratified ON-cell in layer S5 similar to type 9n in EyeWire (Bae et al., 2018). 27.0% (95% CI: 21.3%–32.7%) of GPR88-Cre RGCs demonstrated a morphology similar to the ONdsRGC with dendrites stratifying primarily in layer S4 and a smaller contribution in the upper OFF layer S1 (Bae et al., 2018; Ruff et al., 2021; Wang et al., 2023). In line with our observation of CART-positive RGCs, 18.5% (95% CI: 13.1%–23.9%) of GPR88-Cre RGCs stratified with ChAT-positive amacrine cells in layers S2 and S4 and were thus classified as OOdsRGCs. A smaller proportion (13.0%, 95% CI: 8.3%–17.7%) of GPR88 positive RGCs were monostratified medium-field OFF RGCs in upper S1 that closely resemble type 1no in EyeWire (Bae et al., 2018). The least common RGC found consistently in the GPR88-Cre line (6.0%, 95% CI: 2.7%–9.3%) was a small-field bushy RGC that stratified in prominently in layers S1 with further arborization from S2 to S3, resembling F-mini OFF RGCs (type 2an in EyeWire (Bae et al., 2018; Goetz et al., 2022)). Finally, 15 of the 200 RGCs were observed sporadically, from 1 to 5 times each across the cohort of 200 observed RGCs. Collectively these cells represented 7.5% of the population and were classified as miscellaneous. Taken together, GPR88-Cre provides genetic access to ONdsRGCs and OOdsRGCs, but also targets multiple other RGC types, primarily medium-field monostratified OFF and ON RGCs.

### Brain projections of G-protein-coupled receptor 88 retinal ganglion cells

**Figure 3:**
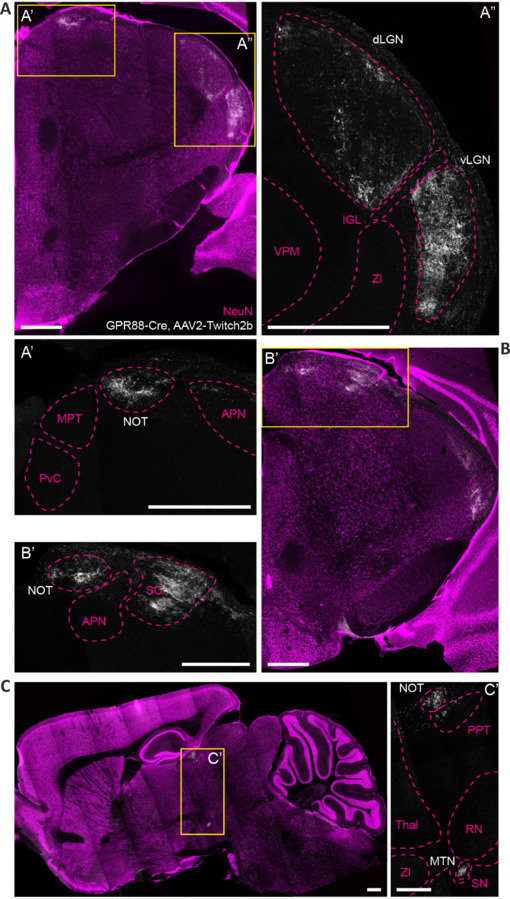
Brain projections of GPR88-Cre RGCs. (A, B) Confocal maximum intensity project of coronal brain slices of GPR88-Cre mice intravitreally injected with AAV2-FLEX-Twitch2b (grayscale) and immunostained for NeuN (magenta). A’, A” and B’ are insets as indicated by yellow boxes. (C) Confocal maximum intensity project of a sagittal brain slice of GPR88-Cre mouse intravitreally injected with AAV2-FLEX-Twitch2b and immunostained for NeuN. C’ is inset as indicated by the yellow box. Scale bars: 500 μm. APN: Anterior pretectal nucleus; dLGN: dorsal lateral geniculate nucleus; GPR88: G-protein-coupled receptor 88; IGL: intergeniculate leaflet; MPT: medial pretectal area; MTN: medial terminal nucleus; NOT: nucleus of the optic tract; PPT: posterior pretectal nucleus; PrC: precommissural nucleus; RGCs: retinal ganglion cells; RN: red nucleus; SC: superior colliculus; SN: substantia nigra; Thal: thalamus; vLGN: ventral lateral geniculate nucleus; VPM: ventral posteromedial nucleus; ZI: zona inserta.

Dozens of brain regions receive input from RGCs, and RGC types project to different brain regions (Baver et al., 2008; Yonehara et al., 2009; Dhande et al., 2013). Thus, we examined which brain regions received inputs from GPR88-Cre RGCs. To specifically label RGCs in GPR88-Cre transgenic mouse brains, we intravitreally injected Cre-dependent AAVs expressing Twitch2b (*n* = 6). After allowing 2–3 weeks for transgene expression, sagittal and coronal brain sections were immunostained for NeuN to identify brain region boundaries and image YFP fluorescence. As expected, GPR88-Cre RGC axons were present in the primary visual areas like the dorsolateral geniculate nucleus and superior colliculus (**Figure 3A** and **B**). Additionally, we observed labeled axons in the ventral LGN, intergeniculate leaflet, nucleus of the optic tract, and the medial terminal nucleus (**Figure 3B** and **C**). The presence of GPR88-Cre labeled axons projecting to the nucleus of the optic tract and medial terminal nucleus aligns with our observation of RGCs with an ONds and OOds morphology as the nucleus of the optic tract and medial terminal nucleus receive retinal inputs from dsRGCs (Yonehara et al., 2009; Dhande et al., 2013). We did not observe RGC axons in the suprachiasmatic nucleus (not shown), indicating that M1 or M2 ipRGCs are unlikely to contribute to the GPR88-Cre population despite the presence of Tbr2 immunopositive RGCs in the GPR88-Cre line (Baver et al., 2008; Chen et al., 2021; **Figure 1G** and **H**).

### Homeostatic Ca^2+^ in G-protein-coupled receptor 88-Cre retinal ganglion cells

We have previously demonstrated that RGCs with higher levels of homeostatic Ca^2+^ are resilient to ONC both within and across RGC types (McCracken et al., 2023). To examine Ca^2+^ levels in GPR88-Cre RGCs we expressed the FRET Ca^2+^ biosensor Twitch2b (Thestrup et al., 2014) using intravitreal injection of Cre-dependent AAV2 expression constructs and *in vivo* transpupillary 2-photon microscopy (Wang et al., 2021; McCracken et al., 2023). We compared GPR88-Cre RGCs to the complete RGC population labeled in VGlut2-Cre transgenic mice (Zhang et al., 2019; McCracken et al., 2023). In accordance with previous observations that showed resilient αRGCs and ipRGCs have higher homeostatic Ca^2+^ levels (McCracken et al., 2023), we observed that GPR88-Cre RGCs also have higher baseline Ca^2+^ levels than all RGCs labeled in the VGlut2-Cre transgenic line (Twitch2b ratio GPR88-Cre 1.19 ± 0.03 *vs.* VGlut2-Cre 1.06 ± 0.01, *P* < 0.0001; **Additional Figure 2A** and **B**). We then performed ONC and tracked RGC survival and Ca^2+^ levels by measuring Twitch2b ratios in GPR88-Cre retinas every 2 days for 14 days after ONC (**Additional Figure 2C**. In line with previous observations, we found that surviving GPR88-Cre RGCs displayed higher baseline Ca^2+^ levels than GPR88-Cre RGCs that died (surviving Twitch2b ratio 1.43 ± 0.07 *vs.* dying 1.10 ± 0.03, *P* = 0.002, **Additional Figure 2D**). To compare survival dynamics, we divided the GPR88-Cre RGC population into equal high- and low-Ca^2+^ halves and compared their survival over the 2-week time lapse. We observed that high-Ca^2+^ GPR88-Cre RGCs survived better 3.2 ± 0.8 fold better (*n* = 268 RGCs from 6 retinas) than low-Ca^2+^ RGCs after ONC. Taken together, these results show that, like other well-surviving RGC types, GPR88-Cre RGCs have a higher distribution of homeostatic Ca^2+^ levels (McCracken et al., 2023), which are correlated with survival within the GPR88-Cre population.

### Resilient G-protein-coupled receptor 88-Cre retinal ganglion cells

**Figure 4:**
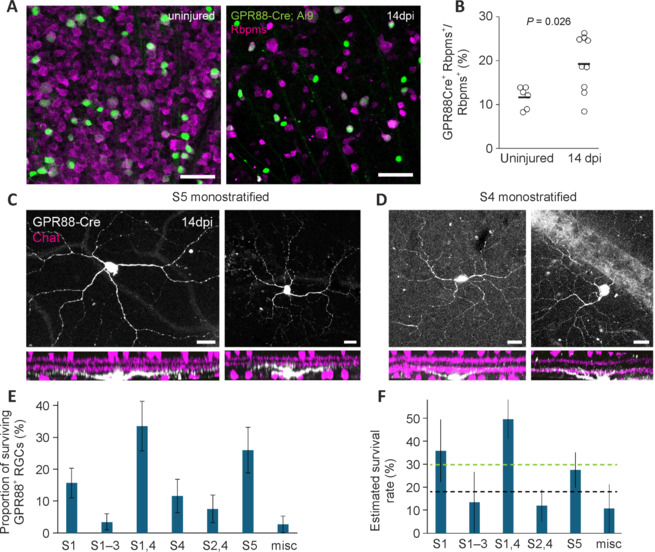
Survival of GPR88-Cre RGCs after ONC. (A) Confocal maximum intensity projections of GPR88-Cre x Ai9 (green) retinal wholemounts that were stained for Rbpms (magenta) before (left) and 14 days after ONC (right). Scale bars: 50 μm. (B) Quantification of the proportion of RGCs that are GPR88-Cre x Ai9 positive in uninjured and 14 dpi retinas. Points indicate individual samples, bars indicate mean. Mann–Whitney *U* test. (C, D) Confocal maximum intensity projections (upper) and orthoslice view (lower) of individually labeled S5 stratifying (C) or S4 stratifying (D) GPR88-Cre RGCs (grayscale) in retinal wholemounts immunostained for ChAT (magenta). Scale bars: 20 μm. (E) Quantification of the morphologically distinct RGC types labeled in GPR88-Cre retinas 14 days after ONC (*n* = 146 RGCs from 11 retinas). Error bars are 95% confidence interval. (F) Estimated true survival rates of individual GPR88-Cre labeled RGC types. Dashed black line indicates all RGCs labeled by Rbpms. Dashed green line indicates GPR88-Cre x Ai9 cohort as a whole. ChAT: Choline acetyltransferase; GPR88: G-protein-coupled receptor 88; ONC: optic nerve crush; RGCs: retinal ganglion cells.

To examine the survival potential of all GPR88-Cre RGCs as a cohort, we performed bilateral ONC in GPR88-Cre; Ai9 transgenic mice and collected retinas for Rbpms immunostaining two weeks later. In line with the idea that GPR88 is a marker for resilient RGCs (Tran et al., 2019), we found that the surviving proportion of GPR88-Cre RGCs post-crush was significantly higher than the native proportion of GPR88-Cre RGCs in the uninjured retina (19.2% ± 2.6% *vs.* 11.6% ± 1.0% respectively, *P* = 0.026; **Figure 4A** and **B**), indicating that, as a group, the five RGC types labeled by GPR88-Cre survive better than RGC the population at large.

However, the GPR88-expressing C10 RGC survives at nearly 4-fold the RGC population average (Tran et al., 2019). Thus, we reasoned that one or more of the individual RGC types labeled in the GPR88-Cre transgenic line likely survive better than other types in the cohort. To determine which of the RGC types that express GPR88 are well-surviving, we labeled RGCs using either low titer injections of AAVs carrying Cre-dependent YFP and mCherry expression constructs in separate injections (88 RGCs from 9 mice), or used complete cohort labeling in GPR88-Cre; Ai9 double transgenic mice (58 RGCs from 3 mice). We performed ONC in these mice, waited 2 weeks for RGC death, and then immunostained retinas for Rbpms to confirm that labeled cells were RGCs, and ChAT to delineate IPL strata (**Figure 4C–E**). High-resolution confocal scans of isolated RGCs were acquired, and RGCs were assigned a type based on morphologies and found the following proportions after ONC (95% CI: S1 9.7%–21.6%; S1–S3 0.1%–5.4%; S2,S4 3.2%–11.8%; S1,S4 25.7%–41.0%, S4 6.4%–16.8%, S5 18.7%–33.0%). Post-ONC distributions showed enrichment of GPR88-Cre RGCs for S1,S4 likely ONdsRGCs (123.5% of uninjured), and S1 OFF (120.3%) monstratified RGCs that were relatively similar to uninjured samples. In line with previous reports of their susceptibility (Duan et al., 2015; Daniel et al., 2018; Tran et al., 2019), S1–S3 presumed F-mini OFF RGCs (4.4%), and S2,S4 presumed OOdsRGCs (40.4%) were decreased relative to uninjured. Interestingly, we also observed a population of monostratified RGCs that projected to S4 not observed in uninjured samples (17 of 146 RGCs; **Figure 4C–E**), which we interpret to be ONdsRGCs that may have retracted their smaller OFF projecting arbors. This interpretation is supported by observations of uninjured retinas where ONdsRGCs labeled by FLRT3-CreERT2 were observed lacking OFF stratifying dendrites in healthy conditions (Ruff et al., 2021). Adding together the S1,S4 and S4 cohorts to represent ONdsRGCs brings their proportion relative to control to 166.3%. Finally, since we observed overall RGC survival rates of 17.9% ± 1.3% by quantifying Rbpms after ONC and GPR88-Cre RGCs survived 65.2% ± 7.5% better than all RGCs, we estimate the following survival rates for our five GPR88-Cre RGC types: S1 35.6%, S1–S3 13.4%, S1,4 49.2%, S2,4 11.9% and S5 RGCs 27.3% (**Figure 4F**). Thus, the ONdsRGCs most likely represent the GPR88-expressing well-surviving RGC type identified in the C10 RGC types in accordance with sequencing studies (Tran et al., 2019; Goetz et al., 2022; Huang et al., 2022).

### Regenerative potential of G-protein-coupled receptor 88-Cre retinal ganglion cells

Different resiliency and regenerative potential are observed across RGC types (Duan et al., 2015; Daniel et al., 2018; Tran et al., 2019; Jacobi et al., 2022). While GPR88-expressing RGCs survive well following optic nerve crush (Tran et al., 2019), the regenerative potential of GPR88-expressing RGCs has not been directly examined. To determine the regenerative potential of GPR88-Cre RGCs, we crossed GPR88-Cre transgenic mice with Ai9 reporter mice, and treated mice with well-characterized pro-regenerative treatments including intravitreal injections of AAV2 carrying short-hairpin RNA targeting PTEN (shPTEN), overexpressing human CNTF (hCNTF), control AAV, or both shPTEN and hCNTF (Park et al., 2008; Smith et al., 2009; Sun et al., 2011; Zukor et al., 2013). After allowing 2–3 weeks for transgene expression, we performed bilateral ONC. Twelve days later, mice received intravitreal injection of Alexa Fluor conjugated CTB to trace axons regenerating in the optic nerve, and mice were perfused 2 days later.

**Figure 5:**
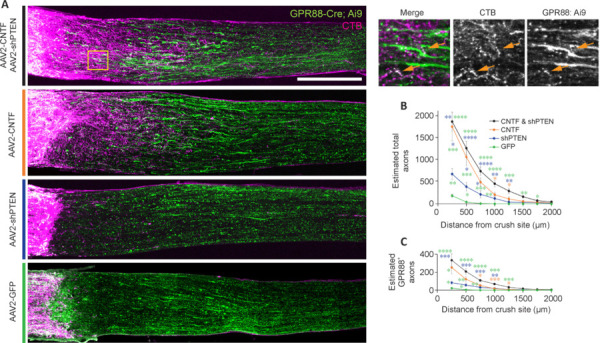
Axon regeneration in GPR88-Cre RGCs. (A) Confocal maximum intensity projections of 12 μm thick optic nerve sections from GPR88-Cre x Ai9 transgenic mice (green) with regenerating axons labeled by intravitreal CTB injection (magenta). Indicated treatment AAVs were delivered 2 weeks before optic nerve crush, and samples were collected 2 weeks later. Insets are from the indicated yellow box. Orange arrows indicate CTB labeled GPR88-Cre x Ai9 axons. Scale bar: 250 μm. (B) Quantification of GPR88-Cre x Ai9 and CTB double-labeled axons regenerating at indicated distances in indicated treatments. (C) Quantification of all CTB labeled RGC axons regenerating at indicated distances in indicated treatments. Two-way analysis of variance with Bonferroni correction, color indicates compared group, *P < 0.05, **P < 0.01, ** *P < 0.01, ****P < 0.001. AAV: Adeno-associated virus; CNTF: ciliary neurotrophic factor; CTB: cholera toxin subunit B; GFP: green fluorescent protein; GPR88: G-protein-coupled receptor 88; RGCs: retinal ganglion cells.

To assess GPR88 RGC axon regeneration, optic nerves were sectioned at 12 μm, and confocal scans of tdTomato labeled GPR88-Cre axons and axon fragments along with CTB labeled regenerating axons were acquired (**Figure 5A**). As expected, regenerative gene therapy treatments lead to significant growth of RGC axons past the crush injury as represented by CTB-positive axons (**Figure 5B**). However, GPR88-Cre expressing RGC axons demonstrated minimal axon regeneration in shPTEN, hCNTF or combinatorial treatment conditions (**Figure 5C**) and accounted for a small proportion of regenerating axons (shPTEN 14.8% ± 1.9%, hCNTF 12.1% ± 2.3%, shPTEN & hCNTF 15.3% ± 0.7% and vehicle 10.9% ± 2.3%).

### Testing protective potential of G-protein-coupled receptor 88 agonists

**Figure 6:**
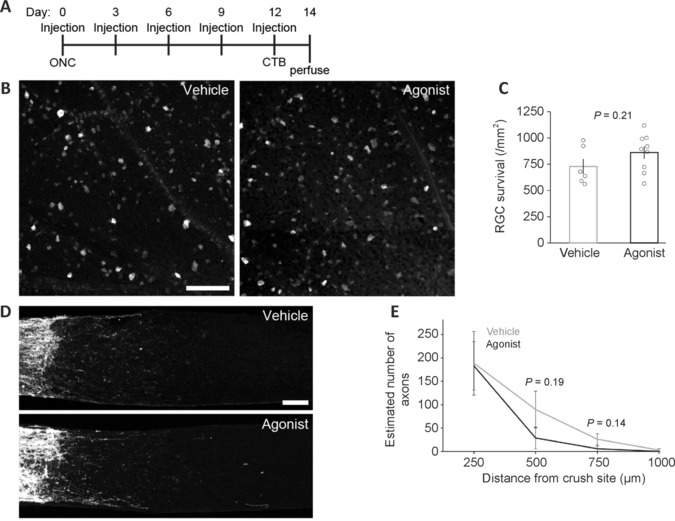
Stimulating GPR88 signaling does not alter RGC response to degeneration. (A) Schematic of ONC and treatment with GPR88 agonist (1R,2R)-2-PCCA or vehicle control. (B) Confocal maximum intensity projections of wholemount retinas following indicated treatments immunostained for Rbpms. (C) Quantification of RGC density in vehicle (*n* = 6 retinas) and (1R,2R)-2-PCCA (*n* = 9 retinas). Data are expressed as mean ± SEM, point = individual samples. Mann–Whitney *U* test. (D) Confocal maximum intensity projections of 14 μm thick optic nerve sections from mice treated as indicated. (E) Quantification of the estimated number of axons at indicated distances in indicated treatments. Analysis of variance with Bonferroni correction. CTB: Cholera toxin subunit B; GPR88: G-protein-coupled receptor 88; RGCs: retinal ganglion cells.

Since some GPR88-expressing RGCs are resilient to ONC, it is possible that GPR88 signaling itself contributes to their higher survival. Thus, we attempted to treat RGCs with a GPR88 agonist following ONC. After performing ONC, we injected the GPR88 agonist (1R,2R)-2-PCCA (Li et al., 2013) immediately following and on days 5 and 9 after injury (**Figure 6A**). On day 12 after ONC, we injected CTB and perfused mice 2 weeks after ONC. To examine the protective effects of GPR88 agonist treatments, we examined RGC density in treated and vehicle control samples. We found that (1R,2R)-2-PCCA treatment did not significantly increase RGC survival (agonist 861.4 ± 59.1 RGCs/mm^2^, vehicle 729.0 ± 72.7 RGCs/mm^2^, *P* = 0.21; **Figure 6B** and **C**). To examine the possibility of axon regeneration after PR88 agonist treatment, we compared axon growth past the lesion between treatment and vehicle control using CTB tracers. We did not observe a difference in the density of CTB-labeled axons at any distance analyzed past the injury site (**Figure 6D** and **E**). Taken together, our results suggest that GPR88 signaling may not directly contribute to RGC survival.

## Discussion

### Retinal ganglion cell types labeled in G-protein-coupled receptor 88-Cre retinas

Our morphological data indicate diverse RGC types are labeled in the GPR88-Cre retina. Roughly 5 prominent morphologically defined types are reliably present. Two of these types match clearly with OOdsRGCs and ONdsRGCs both in their dendritic morphology and expected axonal projections to visual centers in the brain (Yonehara et al., 2009; Dhande et al., 2013). The presence of OOdsRGCs in GPR88-Cre transgenics is in agreement with *in situ* hybridization for GPR88 performed in sparse Thy1 transgenic mice (Tran et al., 2019). Furthermore, experiments aligning electrophysiological, morphological, and transcriptomic characteristics have suggested that the GPR88-expressing C10 RGC is the ONdsRGC (Goetz et al., 2022; Huang et al., 2022). However, given that ONdsRGCs only make up roughly a quarter of RGCs labeled by GPR88-Cre, this may not be the most useful transgenic approach to label these cells given other recent tools like Flrt3-CreERT2 transgenic mice (Ruff et al., 2021). We also identified a small bistratified RGC with morphology aligning to F-mini OFF RGCs (Rousso et al., 2016; Bae et al., 2018; Goetz et al., 2022). The other 2 RGC types in the GPR88-Cre line are less easily identified by morphology alone. Given that we observed ipRGCs labeled by Tbr2 antibodies, we expect that the monostratified ON RGC is the M5 RGC suggested to match with C22 (Tran et al., 2019; Huang et al., 2022), which expresses Tbr2, stratifies in the S5 layer and has a medium arbor size (Sonoda et al., 2020; Chen et al., 2021). However, the monostratified ON-RGC is also similar to the PixON RGC identified as type 9n by EyeWire (Bae et al., 2018; Johnson et al., 2018). The OFF monostratified RGC in the GPR88-Cre line most closely resembles the 1no type from EyeWire (Bae et al., 2018), which demonstrates OFF-sustained responses distinct from the OFF α-sustained RGC and is linked to the C8 RGC (Goetz et al., 2022). We do not believe that the monostratified RGCs in the GPR88-Cre transgenic are alpha, M1, or M2 RGCs. The presence of very few Spp1 immunopositive cells in the GPR88-Cre line indicates few if any αRGCs are labeled and fall within the miscellaneous classification. Further, the dendritic arbor sizes and densities of monostratified GPR88-Cre RGCs are too small and sparse to represent M1 or M2 RGCs (Berson et al., 2002; Schmidt and Kofuji, 2009), and a lack of axon projections to the suprachiasmatic nucleus further supports this conclusion (Baver et al., 2008). Lastly, we observed very few regenerating GPR88-Cre positive axons following shPTEN and CNTF treatment, which should be abundant if αRGC or M1/M2 RGCs were labeled (Duan et al., 2015; Jacobi et al., 2022).

### G-protein-coupled receptor 88 signaling and retinal ganglion cell survival

We were initially surprised that GPR88-Cre RGCs did not display more resilience to ONC (Tran et al., 2019). However, given the diversity of RGC types labeled in the GPR88-Cre line, it was perhaps not surprising that their combined survival would approximate the complete RGC population. Isolating the survival of RGC types identified by single-cell morphological reconstructions roughly aligns each cell type with its survival rate defined by single-cell sequencing (Tran et al., 2019). The ONdsRGC (layers S1 and S4) is the most well surviving of the GPR88-Cre labeled types and corresponds to the well-surviving C10 type. OOdsRGCs are well-documented to survive poorly (Duan et al., 2015; Daniel et al., 2018; Tran et al., 2019; McCracken et al., 2023) corresponding with the poor survival of OOdsRGCs (layers S2 and S4) in the GPR88-Cre line here. The small-field OFF RGC (layers S1–3) that we presume to be the F-mini OFF cell survived below baseline in our experiments with the GPR88-Cre line and with previous reports using both immunostaining and transcriptomics (Tran et al., 2019). The OFF-RGC (layer S1) that we presume to be C8 survived above average in the GPR88-Cre line, and survives at or slightly below average as measured by transcriptomics (Tran et al., 2019). The most discrepant survival result is that of the ON-RGC (layer S5) which we believe to represent the M5 RGC, which has been linked to C22 (Goetz et al., 2022; Huang et al., 2022). In the GPR88-Cre transgenic, the S5 survived near to below average, whereas, by transcriptomics, the C22 survives at double the rate of the population average (Tran et al., 2019). The GRP88-Cre ON monostratified RGC was also morphologically similar to the PixON RGC linked to C41, which survives lower than our observations at roughly half the rate of RGCs as a whole (Tran et al., 2019). It is possible that the M5 may be incorrectly associated with type C22 as this grouping was initially proposed to be the M3 (Tran et al., 2019; Goetz et al., 2022; Huang et al., 2022). Alternatively, GPR88-Cre may label more than one monostratified ON RGC type. Electrophysiological approaches could be applied to discern between these possibilities.

It is perhaps not surprising that treatment with GPR88 agonists did not significantly increase RGC survival. GPR88 is an orphan g-protein coupled receptor (GPCR) that primarily signals through Gi/o proteins and has basal constitutive activity (Jin et al., 2018). GPR88 modulates neurotransmitter receptors and can blunt the function of other stimulating GPCRs (Massart et al., 2009; Meirsman et al., 2016; Laboute et al., 2020). The general inhibitory functions of GPR88 contrast with interventions that activate Gαs signaling like melanopsin, dopamine receptors, and excitatory DREADDs, which all protect RGCs from ONC and promote modest axon regeneration (Li et al., 2016). GPR88 signaling may still be protective, but native signaling could already be present that intermittent agonist treatment does not further stimulate since the native GPR88 ligand is unknown and constitutive activity has been reported for GPR88 (Dzierba et al., 2015). It may then be of interest to convey GPR88 expression to other RGC types that normally do not express the receptor to see if this treatment may convey protective effects.

### Limited axon regeneration

Although many treatments that induce RGC axon regeneration are also protective (Park et al., 2008; Smith et al., 2009; Sun et al., 2011; Duan et al., 2015; Tran et al., 2019; Zhang et al., 2019; Tian et al., 2022), treatments that promote only survival or axon regeneration demonstrate these responses can be uncoupled (Chierzi et al., 1999; Guo et al., 2021; Tian et al., 2022). Most strikingly, boosting CamKII signaling promotes strong RGC survival, but conversely suppresses axon regeneration induced by other pathways like PTEN knockout (Guo et al., 2021; Xia et al., 2024). These pro-survival manipulations are thought to prevent initial injury signaling, and thus prevent cell death cascades but also block initiation of regenerative growth. It is possible that protective but non-regenerative signaling cascades are natively active in ONdsRGCs to maintain their numbers during degeneration but prevent them from regenerating after treatment. More extensive experiments testing the possibility of axon regeneration in ONdsRGCs could determine their regenerative capacity, and may inform future strategies to pair strong RGC protection with axon regeneration (Jacobi et al., 2022).

Different axon regenerative treatments can recruit different RGC populations (Norsworthy et al., 2017; Jacobi et al., 2022). In fact some treatments, like Sox11 overexpression, can kill resilient RGCs in the healthy retina while inducing survival and axon regeneration of otherwise vulnerable types (Norsworthy et al., 2017). We tested the combinatorial shPTEN and hCNTF treatment here because it is one of the most well-characterized axon regeneration treatments (Park et al., 2008; Smith et al., 2009; Sun et al., 2011; Zukor et al., 2013), yet some RGC types regenerating from this treatment remain unidentified (Duan et al., 2015; Jacobi et al., 2022). The limited regeneration we observed in GPR88-Cre RGCs would suggest that the ONdsRGCs are not part of this population.

Great strides have been made to pair single-cell sequencing with paradigms of RGC survival and axon regeneration (Tran et al., 2019; Jacobi et al., 2022). These approaches have identified new extracellular signaling cascades that can regenerate RGC axons including CRH/urocortin and galanin, and have begun to identify which RGC types are well-surviving and regenerating. However, RGC transcriptomes can change drastically after injury and/or regenerative treatments and such analyses can prove difficult to align RGC types across experiments. More direct assessment of RGC type-specific survival and regeneration in defined RGC populations can better inform the diversity of RGC resiliency and regenerative capabilities. Furthermore, more specific genetic access of RGC types with unique and extreme responses to degeneration could be leveraged to further improve the protection and regeneration of neurons in the face of degeneration.

## Limitations

This study has some limitations that should be noted. For one, the GPR88-Cre used here is a BAC transgenic mouse. Although we found colocalization between *GPR88* mRNA and Cre-reporter mouse expression in RGCs, a more precise GPR88-IRES-Cre transgenic may lead to more accurate targeting of ONdsRGCs. Recent reports indicate that BNC2 may be a marker more restricted to ONdsRGCs (Wang et al., 2023), and could thus be used to drive Cre expression for future labelling and manipulation. We report that treatment with GPR88 agonists does not improve RGC survival or axon regeneration; however, drugs delivered to mice via intravitreal injection are challenging to apply repeatedly without causing damage. Perhaps treatments formulated in a suppository with slow release may demonstrate beneficial effects.

## Conclusion

We determined that while GPR88-expressing RGCs are enriched for ONdsRGCs, these are not the only RGCs or retinal neurons present in the GPR88-Cre transgenic mouse. We demonstrate that the ONdsRGC type is in fact well-surviving in degenerative conditions induced by optic nerve injury. In accordance with studies of other resilient RGC types such as αRGCs (Mak et al., 2020), we show that even though ONdsRGCs survive well, their dendritic arbors simplify in response to degeneration. Lastly, we found that ONdsRGCs do not regenerate their axons in response to PTEN knockdown and CNTF overexpression by AAV. Thus, ONdsRGCs may be a fruitful model for unlocking axon regeneration treatments that function more broadly across RGC types.

## Additional files:

***Additional Figure 1:*** *GPR88-Cre recapitulates endogenous expression.*

Additional Figure 1 GPR88-Cre recapitulates endogenous expression.
(A) Confocal maximum intensity projection of GPR88-Cre x Ai9 retinal section stained with *in situ* hybridization probes for endogenous *GPR88* (green) and the *tdTomato* transgenic reporter (magenta) counterstained with DAPI (blue). Dashed orange box shows region magnified in insets. (B) Quantification of the proportion of *GPR88^+^* cells that were also *tdTomato^+^*, and *tdTomato^+^* cells that were *GPR88^+^*.

***Additional Figure 2:*** *Surviving GPR88-Cre RGCs demonstrate higher Ca*^*2+*^ *levels.*

Additional Figure 2 Surviving GPR88-Cre RGCs demonstrate higher Ca^2+^ levels.
(A) Maximum intensity projections of *in vivo* 2-photon stacks of the Ca^2+^ biosensor Twitch2b expressed in all RGCs (VGlut2-Cre, left) or GPR88-Cre RGCs (right). Scale bar: 50 μm. (B) Quantification of Ca^2+^ levels in indicated RGC groups (*n* = 533 RGCs from 9 retinas VGlut2-Cre, n = 252 RGCs from 6 retinas GPR88-Cre). Points indicate individual RGCs and bars indicate sample mean. Mann-Whitney *U* test. (C) Time series of maximum intensity projections of *in vivo* 2-photon stacks of GPR88-Cre retinas injected with AAV2-FLEX-Twitch2b at indicated time points relative to ONC. Arrows indicate surviving higher Ca^2+^ cells. (D) Quantification of baseline preinjury Ca^2+^ levels in GPR88-Cre RGCs that survived or died after ONC. Points indicate individual RGCs and bars indicate sample mean (*n* = 268 RGCs from 6 retinas). Mann-Whitney *U* test.

## Data Availability

*All relevant data are within the paper and its Additional files*.
