# Supplementary figures and images for: Identification of a natively resilient but poorly regenerating retinal ganglion cell type in the G protein-coupled receptor 88-Cre transgenic mouse
Source: Neural Regen Res. 2025 Aug 13;21(7):3194–201. doi: 10.4103/NRR.NRR-D-24-01270 (PMC13378955; doi:10.4103/NRR.NRR-D-24-01270)

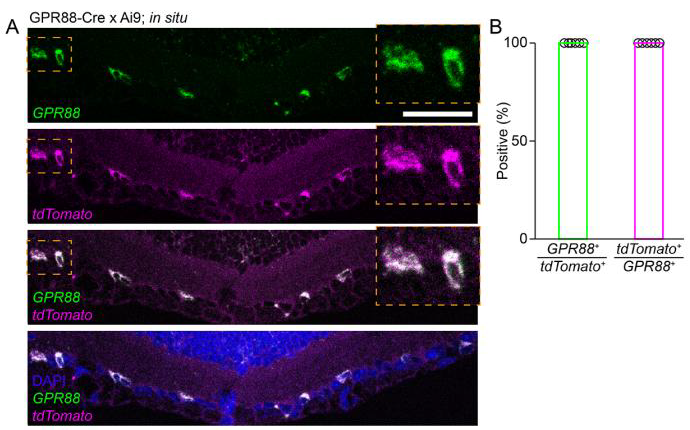

Supplement: Supplementary file 1 [file NRR-21-3194_Suppl1.tif]

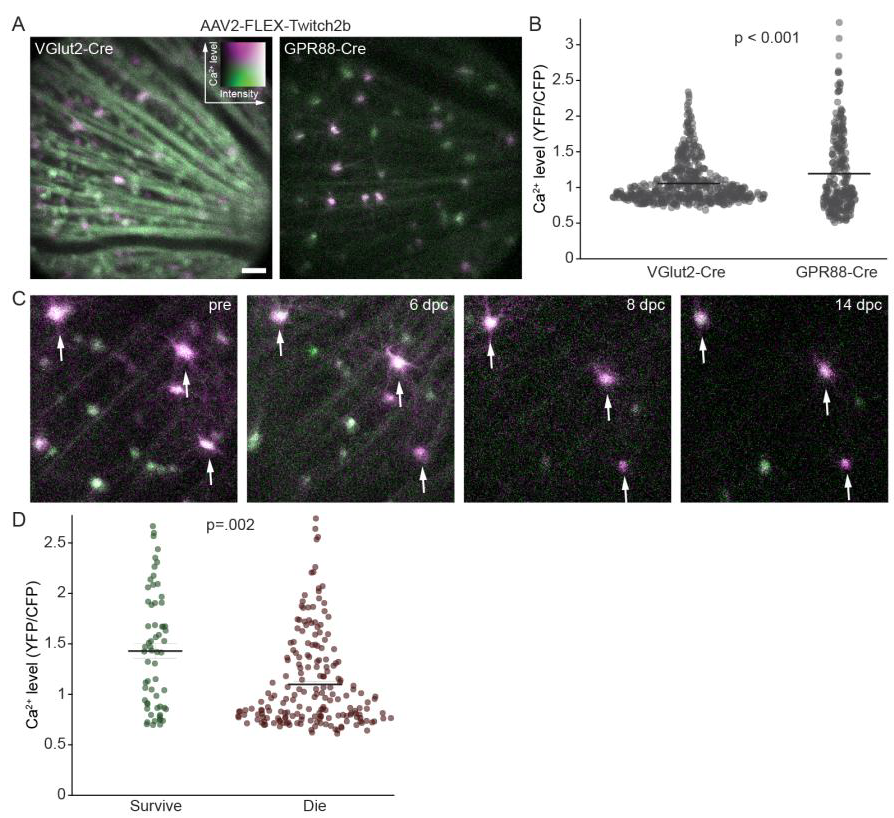

Supplement: Supplementary file 2 [file NRR-21-3194_Suppl2.tif]
